# Comparison of L1 and L5 Bands GNSS Signals Acquisition

**DOI:** 10.3390/s18092779

**Published:** 2018-08-23

**Authors:** Jérôme Leclère, René Landry, Cyril Botteron

**Affiliations:** 1 LASSENA, École de Technologie Supérieure (ÉTS), 1100 Notre-Dame Street West, Montreal, QC H3C 1K3, Canada; renejr.landry@etsmtl.ca; 2 Syderal, Neuenburgstrasse 7, 3238 Gals, Switzerland; cyril.botteron@syderal.ch

**Keywords:** acquisition, complexity, Galileo, GPS, GNSS, hardware receiver

## Abstract

Nowadays, civil Global Navigation Satellite System (GNSS) signals are available in both L1 and L5 bands. A receiver does not need to acquire independently the signals in both bands coming from a same satellite, since their carrier Doppler and code delay are closely related. Therefore, the question of which one to acquire first rises naturally. Although the common thought would tell the L1 band signals which are narrowband, an accurate comparison has never been done, and the decision is not as easy as it seems. Indeed, L5 band signals have several advantages such as stronger power, lower carrier Doppler, or a pilot channel, unlike the Global Positioning System (GPS) L1 C/A signal. The goal of this paper is therefore to compare the acquisition of L1 and L5 bands signals (GPS L1 C/A and L5, Galileo E1 and E5a/b) to determine which one is more complex and by which factor, in terms of processing time and memory, considering hardware receivers and the parallel code search. The results show that overall the L5 band signals are more complex to acquire, but it depends strongly on the conditions. The E5 signal is always more complex to acquire than E1, while the L5 signal can have a complexity close to the L1 C/A in some cases. Moreover, precise assistance providing accurate Doppler could significantly reduce the L5 complexity below the L1 complexity.

## 1. Introduction

The first stage of a Global Navigation Satellite System (GNSS) receiver is the acquisition, whose aim is to detect the signal and roughly estimate the code delay and the carrier frequency [1]. This is a computationally demanding operation since there are numerous possibilities to test, and today’s receivers are targeting higher and higher sensitivities and the ability to process more and more signals. Nowadays, Fast Fourier Transforms (FFT) are omnipresent in acquisition architectures to accelerate the acquisition, and the amount of memory needed is a major factor in a design [2].

There are now several signals available per constellation, and it is not necessary to acquire the different signals coming from one satellite independently. Indeed, considering two signals coming from the same satellite (for example Global Positioning System (GPS) L1 C/A and L5 signals): They are synchronized (the primary codes start at the same time, and the data and secondary code transitions are synchronized) [3]. The path traveled is the same, therefore the code delay is about the same (there is a slight difference due to the ionosphere that affects them differently [4]. However, knowing one gives precious information on the second); the relative speed being the same, the Doppler are proportional with a known factor (even with the offset due to the local oscillator). Therefore, the main question is: “Which signal should be acquired first?”, to then help the acquisition of the other(s) signal(s). This paper aims to answer this question, and quantify it with application examples.

The answer to this question is not so simple, because each signal has its own advantages and drawbacks. For example, L5 band signals, such as the GPS L5 and Galileo E5 signals, have a high chipping rate (10 times higher than L1 frequency signals). This high chipping rate implies a high sampling frequency, which implies itself a significant amount of samples to store and process, and a lower ratio between the clock frequency of the acquisition process and the sampling frequency leading to a longer processing time [5]. Therefore, L1 band signals, such as the GPS L1 C/A and Galileo E1 signals, are much more interesting on this side, since there is potentially a factor five or ten in the number of samples to process and in the processing speed, thanks to the ratio of the processing and sampling frequencies. Moreover, the L5 and E5 signals have longer primary codes than the L1 C/A and E1 signals, which a priori implies the testing of more code delays, larger FFTs for the correlations, and a bigger amount of memory to store correlation results.

However, the L5 band signals also have advantages: (1) They have a higher power (1.5 dB for the pilot channel of the L5 signal compared to the L1 C/A signal, and 2 dB for the E5a/b signals compared to the E1 signal for the pilot channels), which means that similar detection performance can be obtained with lower integration times; (2) They have a pilot channel, whereas the GPS L1 C/A signal does not have one, therefore this lasts one must limit its coherent integration time, which can lead to a longer total integration time; (3) They have a lower carrier Doppler and Doppler rate (115/154 ≈ 75%), which can reduce the search space and some constraints for the acquisition architecture; and (4) They have a secondary code, which on one side complicates the acquisition, but on another side makes the data synchronization much easier, simplifying the transition to the tracking.

The goal of this paper is therefore to compare in detail the acquisition of the L1 and L5 bands signals, in terms of processing time and memory requirements. More specifically, the GPS L1 C/A and L5 signals, and the Galileo E1 and E5 signals will be considered since these are the only signals in these two bands defined in an official interface specification and broadcasted currently. Two cases will be considered, one where there is no assistance at all (equivalent to a cold start), and one there is enough assistance to determine the secondary code delay, such that the receiver does not need to search it (kind of warm start). Indeed, it is relatively easy to have an estimate of the current secondary code chip. One chip of the L5 or E5 secondary codes lasts 1 ms, which is equivalent to 300 km. Therefore, if the receiver has an estimate of the current time better than 1 ms and an estimate of its position and of the satellites position better than 300 km (achievable with almanac), it is possible to estimate the current secondary code chip, and thus there is no need to search it via a correlation. If an estimate is available but not so accurate, the number of possibilities can still be reduced, e.g., to three or four, instead of 20 or 100.

The remainder of the paper is organized as follows. Section 2 describes briefly the GNSS signals considered, summarizes their characteristics, and introduces the acquisition search method chosen (parallel code search) and the elements of interest. Section 3 provides mathematical expressions of the processing time and the memory requirements for all the possible implementations: Data channel verses pilot channel, assistance verses no assistance, coherent integration only verses use of non-coherent integration, parallel verses semi-parallel verses serial implementation. Then, Section 4 describes the methodology used for the comparison, details all the parameters selected, and compares the acquisition of the GPS L1 C/A and L5 signals, and the acquisition of the Galileo E1 and E5 signals by evaluating the expressions given in Section 3. Finally, Section 5 summarizes the outcomes.

## 2. GNSS Signals and Acquisition

### 2.1. GNSS Signals

GNSS signals contain three essential components, namely navigation data, a pseudorandom code (also called primary code with the advent of modern signals) and a carrier. Modern signals may also contain other optional elements, such as a pilot channel where there is no random data; a secondary code, helping the data synchronization, reducing cross-correlation, and mitigating interference; and a sub-carrier, for multipath mitigation [6,7]. Table 1 summarizes the characteristics of the GNSS signals considered.

The signal after the front-end of a receiver is a combination of several GNSS signals and noise:

(1) $$s_{b}\left(nT_{S}\right)=\overset{U}{\underset{u=1}{\sum }}s_{b}^{u}\left(nT_{S}\right)+\eta_{b}\left(nT_{S}\right),$$

where $s_{b}^{u}$ is the discrete baseband signal from satellite u, n is the discrete time index, $T_{S}$ is the sampling period equal to $1/f_{S}$ with $f_{S}$ the sampling frequency, and $\eta_{b}$ is a white Gaussian noise.

The signal from one satellite can be expressed in a generic way as

(2) $$\begin{array}{l} s_{b}^{u}\left(nT_{S}\right)=a_{d}^{u}c_{2,d}^{u}\left(nT_{S}-\tau^{u}\right)c_{1,d}^{u}(nT_{S}\\ -\tau^{u})sc_{d}^{u}\left(nT_{S}-\tau^{u}\right)d^{u}\left(nT_{S}-\tau^{u}\right)\cos\left(2\pi \left(f_{\mathrm{IF}}+f_{D}^{u}+f_{\mathrm{OSC}}\right)nT_{S}+\varphi_{d}^{u}\right)\\ +a_{p}^{u}c_{2,p}^{u}\left(nT_{S}-\tau^{u}\right)c_{1,p}^{u}\left(nT_{S}-\tau^{u}\right)sc_{p}^{u}\left(nT_{S}-\tau^{u}\right)\sin\left(2\pi \left(f_{\mathrm{IF}}+f_{D}^{u}+f_{\mathrm{OSC}}\right)nT_{S}+\varphi_{p}^{u}\right), \end{array}$$

where the subscript d and p denotes the data and pilot channels respectively, $a^{u}$ is the amplitude, $c_{1}^{u}$ is the primary code, $c_{2}^{u}$ is the secondary code, $sc^{u}$ is the subcarrier, $d^{u}$ is the navigation data, $\tau^{u}$ is the unknown code delay, $f_{D}^{u}$ is the unknown Doppler frequency, $f_{\mathrm{OSC}}$ is the oscillator offset (not known in a cold start, but may be known or have a good estimation in warm and hot starts [8]), $f_{\mathrm{IF}}$ is the intermediate frequency, and $\varphi^{u}$ is the carrier phase. Note that in Equation (2), the data channel is denoted with a cos and the pilot channel is denoted with a sin, however it does not mean that they are necessary in quadrature, as shown in Table 1; it depends on the value of $\varphi_{d}$ and $\varphi_{p}$ [9]. Furthermore, the code Doppler is not considered in this model to keep it simple. Next, it is assumed that the code Doppler is compensated in the receiver. Since it is proportional to the carrier Doppler, it is relatively simple to adapt the local code chipping rate or to shift the correlation results during their accumulation.

Next, only the pilot channels are considered for the acquisition (except for the GPS L1 C/A signal that has only a data channel), because they allow longer coherent integration time, and it is known that the longer the coherent integration time the better the sensitivity [8,10]. However, it is not always possible to extend the coherent integration time to very long duration, due to the instability of the local oscillator [11,12,13] or the unknown dynamics of the receiver [8,14]. Therefore, to be as general as possible, two cases will be considered for the signal: The first with coherent integration only, without any limit; and the second with a coherent integration of one secondary code period, i.e., 20 ms for the L5 signal and 100 ms for Galileo signals, followed by further non-coherent integration.

### 2.2. Acquisition of GNSS Signals

There are several methods to perform the acquisition of GNSS signals, namely the serial search [1]; the Parallel Frequency Search (PFS), which uses an FFT to search simultaneously all or a part of the Doppler frequency bins [15,16]; the Parallel Code Search (PCS), which uses FFTs to compute the code correlation in order to search simultaneously all the code delay bins [1,17]; and two-dimensional search where FFTs are used for both frequency and code domains [9,16,18,19].

The method considered in this paper is the PCS that computes the correlation over the primary code correlation using FFTs, because [5]: (1) It is an efficient method providing a high level of parallelism; (2) the parallelism is still exploited in presence of a basic assistance, unlike the parallel frequency search (because it is much easier to reduce the frequency search space than the code delay search space from any assistance [8]); (3) the code Doppler can easily be compensated; and (4) it requires less memory than two-dimensional searches. To emphasize this, one can note that this is the method implemented in today’s mass market receivers [2], and considered or already implemented in spaceborne GNSS receivers [20,21,22].

**Figure 1:**
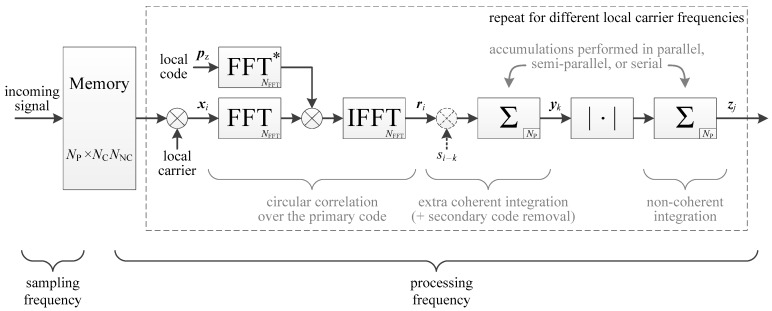
Overview of the considered parallel code search with long integration. Dashed blocks are optional.

An overview of the PCS architecture considered is shown in Figure 1. The incoming signal is stored into a memory, to be processed at a higher frequency in order to reduce the processing time [5]. Then, during the processing, the signal is multiplied by a local carrier, followed by an FFT-based correlation performed over one primary code period (in fact, two primary code periods are used to manage the possible transition and the zero-padding possibly needed, but this is repeated for every period [23,24]). At this stage, if the Doppler is completely removed, the i th primary code correlation result can be expressed as

(3) $$r_{i}=a\;c_{2,i-m_{S}}\;r_{P}+\eta_{i},$$

for a pilot channel signal, or as

(4) $$r_{i}=a\;d_{i}\;r_{P}+\eta_{i},$$

for a data channel signal, where $c_{2,i-m_{S}}$ is the $\left(i-m_{S}\right)$ th chip of the secondary code (the subscript is modulo $N_{S}$, with $N_{S}$ the length of the secondary code in chip), $m_{S}$ is the unknown delay of the incoming secondary code, $r_{P}$ is the primary code autocorrelation of length $N_{P}$ ($N_{P}$ being the number of samples in one primary code period), and $\eta_{i}$ is the noise [23].

Then, further coherent integration can be performed. If there is data, the data transition should be managed; if there is a secondary code, the secondary code should be removed with another correlation. This is described in detail in Section 3. Finally, non-coherent integration can be performed. The number of accumulators used for the coherent and non-coherent integrations can vary depending if the accumulations are performed in serial (one accumulator testing one delay at once), parallel (many accumulators testing all delays at once), or semi-parallel (several accumulators testing several delays at once).

For the comparisons in this paper, the implementations will be based on Figure 1 and inspired from [23], which compares many ways to implement the acquisition (e.g., the accumulation can be done before or after the FFTs, a temporary memory can be used or not, and the accumulation can be done serially or in parallel). The next section will detail the specific cases that interest us, i.e., using coherent integration only or using non-coherent integrations, and considering parallel, serial and semi-parallel accumulations. It is considered that the accumulations are performed after the FFT-based primary code correlation, and that there is no temporary memory since it is useless for coherent integration only and with non-coherent accumulations the structure would become different than the one of the implementations of the L1 C/A signal.

To assess the performance of the different implementations, the processing time and the amount of memory will be evaluated and compared. Therefore, in Figure 1, our focus is on the main memory storing the incoming signal, and on the accumulators after the FFT-based primary code correlation. The primary code correlation is considered traditional, because any method to make it faster such as those proposed in Reference [24,25,26] could be applied to any of the following implementations, and thus would not affect the comparison. Likewise for the secondary code correlation, where there are known methods to reduce the complexity by a factor two at least [27,28,29]. Thus, these methods are not considered in the evaluation of the memory and processing time in Section 3.

## 3. Assessment of Processing Time and Memory Requirements

This section aims to determine the expression of the processing time and of the amount of memory for the different implementations. For this, it discusses first the acquisition of a data channel, and then the acquisition of a pilot channel, without assistance (where the correlation over the secondary code should be performed) and with assistance (where the delay of the secondary code is known, allowing a simple secondary code removal). For each implementation, the timing diagram is provided and the processing time is directly deduced from it, as done in Reference [17,23]. Note that these expressions are fully deterministic and exact since the processing time for one frequency bin is given, not the mean acquisition time [30], which is sufficient to compare the implementations.

### 3.1. Acquisition of a Data Channel

This section discusses the acquisition of a data channel. This mainly concerns the GPS L1 C/A signal, since all the modern signals have a pilot channel.

**Figure 2:**
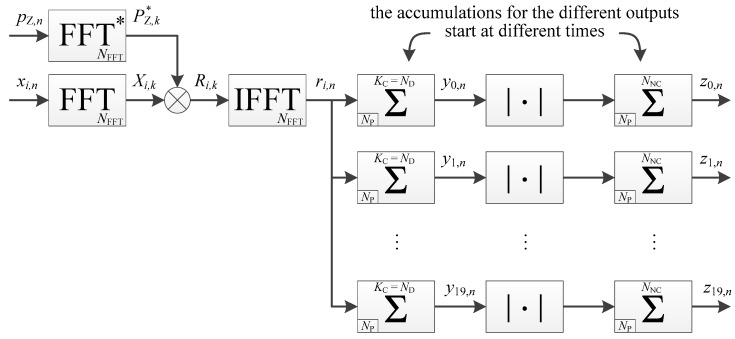
Implementation of a data channel acquisition (timing diagram available in Figure 3).

There are different methods to manage the data transition during the acquisition. Since high sensitivity is considered, the longest coherent integration time is desired. Therefore, the method that computes many accumulations in parallel starting at different times to test all the possibilities, called full-bits method with estimation of bit transition times in Reference [31], is considered. The highest accumulated value should be the one where the accumulation starts with the data bit. Consequently, the implementation would be the one of Figure 2. The size of the memory-based accumulators is $N_{P}=f_{S}\;T_{P}$, with $T_{P}$ the period of the PRN code, e.g., 1 ms for the L1 C/A signal.

The size of the FFT can be $N_{\mathrm{FFT}}=N_{P}$ or nextpow2(2$N_{P}$), where nextpow2(*n*) means the power of two equal to or higher than *n*, i.e., $2^{⎡\log(n)⎤}$. To preserve the periodicity of the code, either the sampling frequency should give a length $N_{P}$ that is a power of two, or the length should be doubled by zero-padding. To avoid the loss due to the data transition within the correlation, the length should be doubled by zero-padding. Therefore, to be as general as possible and consider a flexible choice on the sampling frequency, it will not be assumed that $N_{P}$ is a power of two, and therefore $N_{\mathrm{FFT}}=$ nextpow2(2$N_{P}$)$=2^{⎡\log(2N_{P})⎤}$.

**Figure 3:**
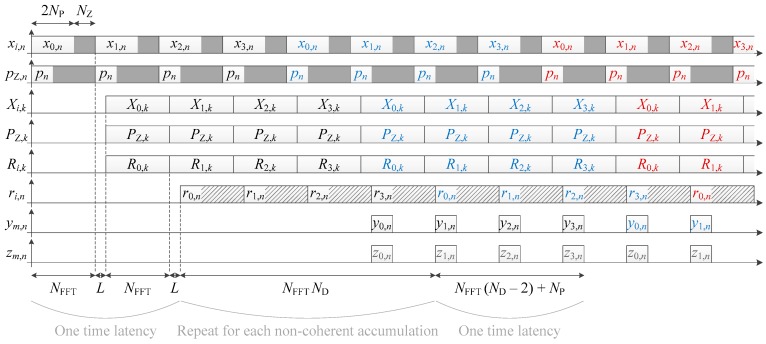
Timing diagram of a data channel acquisition shown in Figure 2, with $N_{D}=4$ for the illustration (in reality $N_{D}=20$ for the GPS L1 C/A signal). Colors indicate different portions corresponding to the length of one data bit. Grey color indicates intermediate results (i.e., the value is not yet the final one).

The corresponding timing diagram is shown in Figure 3, from which we can deduce the processing time in clock cycle for one frequency bin:

(5) $$\begin{array}{l} T_{\mathrm{FB}}=KN_{\mathrm{FFT}}N_{D}+2\left(N_{\mathrm{FFT}}+L\right)+N_{\mathrm{FFT}}\left(N_{D}-2\right)+N_{P}\\ =KN_{D}\left(2N_{P}+N_{Z}\right)+N_{P}\left(2N_{D}-1\right)+N_{Z}N_{D}+2L\\ =N_{P}\left[\left(K+1\right)2N_{D}+1\right]+N_{Z}\left[\left(K+1\right)N_{D}\right]+2L, \end{array}$$

where K is the number of data bits accumulated non-coherently (i.e., the number of non-coherent accumulations here: $K=N_{\mathrm{NC}}$), $N_{\mathrm{FFT}}=2N_{P}+N_{Z}$ with $N_{Z}$ the number of zeros padded, $N_{D}$ is the number of primary code periods in one data bit ($N_{D}=20$ for the GPS L1 C/A signal), and L the latency of the FFT (i.e., the number of clock cycles between the first output sample and the last input sample).

Regarding the amount of memory necessary, the memory-based accumulators require

(6) $$\begin{array}{l} M=N_{D}2N_{P}B_{C}+N_{D}N_{P}B_{\mathrm{NC}}\;\mathrm{bits}\\ =N_{D}N_{P}\left(2B_{C}+B_{\mathrm{NC}}\right)\;\mathrm{bits}\\ =N_{D}N_{P}\left[3B_{R}+3⎡{\log\;}_{2}\left(N_{D}\right)⎤+⎡\log_{2}\left(K\right)⎤\right]\;\mathrm{bits}, \end{array}$$

where $B_{C}$ is the number of bits used to quantize the output of the coherent accumulators ($y_{i}$) and $B_{\mathrm{NC}}$ is the number of bits used to quantize the output of the non-coherent accumulators ($z_{i}$), with

(7) $$B_{C}=B_{R}+⎡{\log\;}_{2}\left(N_{D}\right)⎤,$$

and

(8) $$\begin{array}{l} B_{\mathrm{NC}}=B_{C}+⎡\log_{2}\left(K\right)⎤\\ =B_{R}+⎡{\log\;}_{2}\left(N_{D}\right)⎤+⎡\log_{2}\left(K\right)⎤, \end{array}$$

where $B_{R}$ is the number of bits used to quantize the output of the correlation ($r_{i}$). Note that these last two equations assume that there is no truncation during the accumulations.

### 3.2. Acquisition of a Pilot Channel without Assistance

#### 3.2.1. Coherent Integration Only with Parallel Implementation

As mentioned previously, in the case where there is no non-coherent accumulation, there is only one stage of accumulator-based memory, and therefore there is no interest in having a temporary memory between the Inverse FFT (IFFT) output and the coherent accumulator(s).

**Figure 4:**
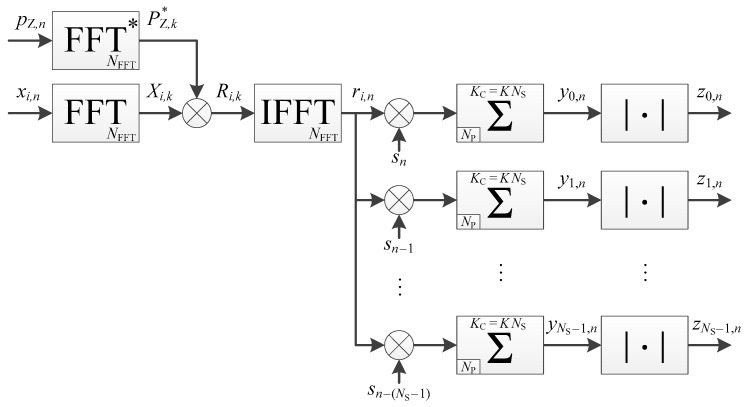
Implementation of a pilot channel acquisition, with coherent integration only, in a parallel way (timing diagram available in Figure 5).

**Figure 5:**
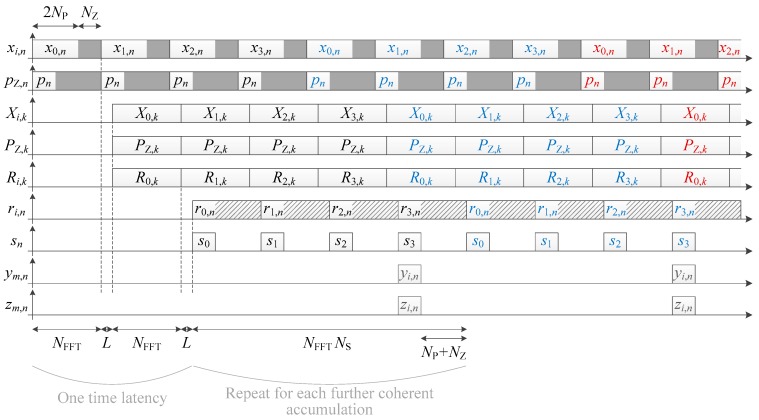
Timing diagram of the pilot channel acquisition shown in Figure 4, with $N_{S}=4$ for the illustration.

The parallel implementation is already given in Reference [23], and shown in Figure 4 (where it is assumed that the coherent integration time is a multiple of the secondary code period), and the corresponding timing diagram is shown in Figure 5. The processing time for one frequency bin is thus

(9) $$\begin{array}{l} T_{\mathrm{FB}}=KN_{\mathrm{FFT}}N_{S}+2\left(N_{\mathrm{FFT}}+L\right)-\left(N_{P}+N_{Z}\right)\\ =KN_{S}\left(2N_{P}+N_{Z}\right)+3N_{P}+N_{Z}+2L\\ =N_{P}\left(2KN_{S}+3\right)+N_{Z}\left(KN_{S}+1\right)+2L, \end{array}$$

where K is the number of secondary code periods accumulated coherently. Thus, $K_{C}=KN_{S}$ correlation results are coherently accumulated (or written in another way, the coherent integration time is $T_{C}=K_{C}T_{P}=KN_{S}T_{P}$).

Regarding the amount of memory necessary, the memory-based accumulators require

(10) $$\begin{array}{l} M=N_{S}2N_{P}B_{C}\;\mathrm{bits}\\ =N_{S}2N_{P}\left(B_{R}+⎡{\log\;}_{2}\left(KN_{S}\right)⎤\right)\;\mathrm{bits}, \end{array}$$

where $B_{C}=B_{R}+⎡\log_{2}\left(KN_{S}\right)⎤$ is the number of bits used to quantize the output of the coherent accumulators ($y_{i}$).

#### 3.2.2. Coherent Integration Only with Serial Implementation

**Figure 6:**
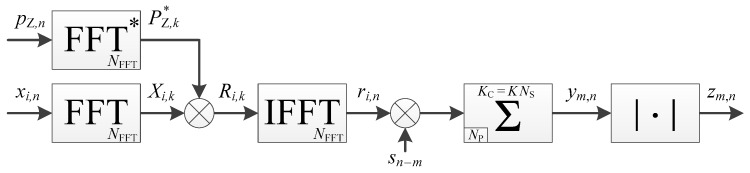
Implementation of a pilot channel acquisition, without non-coherent accumulation, in a serial way (timing diagram available in Figure 7).

**Figure 7:**
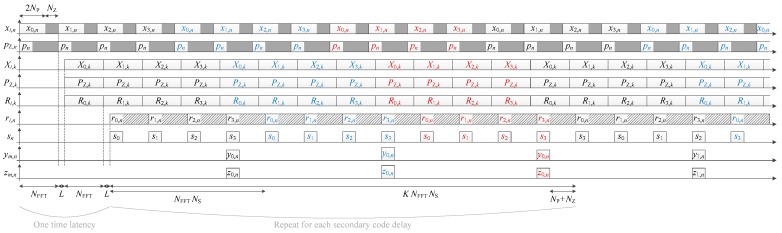
Timing diagram of the pilot channel acquisition shown in Figure 6, with $N_{S}=4$ and K=3.

The serial implementation is already given in Reference [23], and shown in Figure 6, with its corresponding timing diagram in Figure 7. However, the timing diagram in Reference [23] is not totally correct because it shows that the coherent integration for different secondary code delay is computed first and then non-coherent accumulations can be done, which is not correct or would require additional storage. In Figure 7, the correlations and further coherent accumulations are computed first, and then the operation is repeated for different delays of the secondary code. Note, however, that the processing time in Reference [23] is correct and identical to the one given below.

The processing time for one frequency bin is thus,

(11) $$\begin{array}{l} T_{\mathrm{FB}}=KN_{\mathrm{FFT}}N_{S}^{2}+2\left(N_{\mathrm{FFT}}+L\right)-\left(N_{P}+N_{Z}\right)\\ =KN_{S}^{2}\left(2N_{P}+N_{Z}\right)+3N_{P}+N_{Z}+2L\\ =N_{P}\left(2KN_{S}^{2}+3\right)+N_{Z}\left(KN_{S}^{2}+1\right)+2L. \end{array}$$

Regarding the amount of memory necessary, the memory-based accumulator requires

(12) $$\begin{array}{l} M=2N_{P}B_{C}\;\mathrm{bits},\\ =2N_{P}\left(B_{R}+⎡{\log\;}_{2}\left(KN_{S}\right)⎤\right)\;\mathrm{bits}, \end{array}$$

where $B_{C}=B_{R}+⎡\log_{2}\left(KN_{S}\right)⎤$ as previously.

#### 3.2.3. Coherent Integration only with Semi-Parallel Implementation

From the previous cases, it is easy to generalize to the case where $N_{A}$ accumulators tests $N_{A}$ delays in parallel. The processing time for one frequency bin is

(13) $$\begin{array}{l} T_{\mathrm{FB}}=KN_{\mathrm{FFT}}N_{S}⎡\frac{N_{S}}{N_{A}}⎤+2\left(N_{\mathrm{FFT}}+L\right)-\left(N_{P}+N_{Z}\right)\\ =KN_{S}⎡\frac{N_{S}}{N_{A}}⎤\left(2N_{P}+N_{Z}\right)+3N_{P}+N_{Z}+2L\\ =KN_{S}⎡\frac{N_{S}}{N_{A}}⎤\left(2N_{P}+N_{Z}\right)+3N_{P}+N_{Z}+2L\\ =N_{P}\left(2KN_{S}⎡\frac{N_{S}}{N_{A}}⎤+3\right)+N_{Z}\left(KN_{S}⎡\frac{N_{S}}{N_{A}}⎤+1\right)+2L. \end{array}$$

It can be checked that Equation (13) becomes Equation (9), with $N_{A}=N_{S}$, and becomes Equation (11) with $N_{A}=1$.

Regarding the amount of memory necessary, the memory-based accumulators require

(14) $$\begin{array}{l} M=N_{A}2N_{P}B_{C}\;\mathrm{bits},\\ =N_{A}2N_{P}\left(B_{R}+⎡{\log\;}_{2}\left(KN_{S}\right)⎤\right)\;\mathrm{bits}, \end{array}$$

where $B_{C}=B_{R}+⎡\log_{2}\left(KN_{S}\right)⎤$ as previously. In the same way, Equation (14) becomes Equation (10), with $N_{A}=N_{S}$, and becomes Equation (12) with $N_{A}=1$.

#### 3.2.4. Use of Non-Coherent Accumulation with Parallel Implementation

This implementation is very similar to the one of the data channel acquisition; there are only two differences:

- The number of branches is $N_{S}$ instead of $N_{D}$.
- For a pilot channel, the coherent accumulations for the different delays start and finish at the same time, therefore the latency and the length of data needed is slightly reduced.

**Figure 8:**
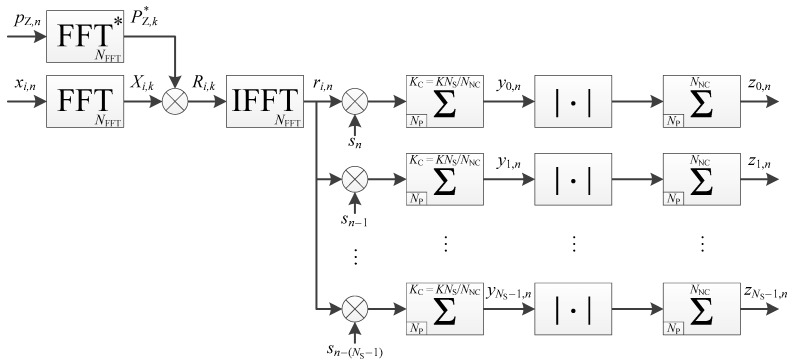
Implementation of a pilot channel acquisition, with non-coherent accumulation, in a parallel way (timing diagram available in Figure 5).

The implementation is shown in Figure 8. Since the accumulations start and finish at the same time, the timing diagram is the same as for the case of coherent integration only, shown in Figure 5, except that the portion repeated is for further coherent and non-coherent accumulations (and the number of repetition is still K).

Regarding the memory, the memory-based accumulators require

(15) $$\begin{array}{l} M=N_{S}2N_{P}B_{C}+N_{S}N_{P}B_{\mathrm{NC}}\;\mathrm{bits}\\ =N_{S}N_{P}\left(2B_{C}+B_{\mathrm{NC}}\right)\;\mathrm{bits}\\ =N_{S}N_{P}\left[3B_{R}+3⎡{\log\;}_{2}\left(KN_{S}/N_{\mathrm{NC}}\right)⎤+⎡\log_{2}\left(N_{\mathrm{NC}}\right)⎤\right]\;\mathrm{bits}, \end{array}$$

with

(16) $$B_{C}=B_{R}+⎡{\log\;}_{2}\left(KN_{S}/N_{\mathrm{NC}}\right)⎤,$$

and

(17) $$\begin{array}{l} B_{\mathrm{NC}}=B_{C}+⎡\log_{2}\left(N_{\mathrm{NC}}\right)⎤\\ =B_{R}+⎡{\log\;}_{2}\left(KN_{S}/N_{\mathrm{NC}}\right)⎤+⎡\log_{2}\left(N_{\mathrm{NC}}\right)⎤. \end{array}$$

#### 3.2.5. Use of Non-Coherent Accumulation with Serial Implementation

**Figure 9:**
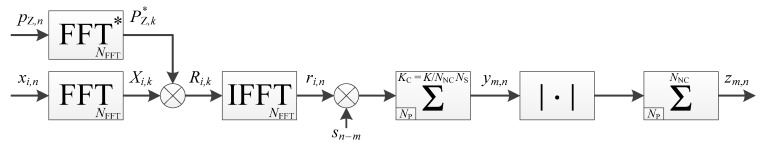
Implementation of a pilot channel acquisition, with non-coherent accumulation, in a serial way (timing diagram available in Figure 7).

This implementation is shown in Figure 9. The processing time is the same as the serial implementation with coherent integration only (Figure 6), because the timing diagram (Figure 7) is similar. Indeed, the number of secondary code periods processed is still $K/N_{\mathrm{NC}}\times N_{\mathrm{NC}}=K$, even if it is spread between coherent and non-coherent accumulations.

Regarding the memory, the memory-based accumulators require

(18) $$\begin{array}{l} M=2N_{P}B_{C}+N_{P}B_{\mathrm{NC}}\;\mathrm{bits}\\ =N_{P}\left(2B_{C}+B_{\mathrm{NC}}\right)\;\mathrm{bits}\\ =N_{P}\left[3B_{R}+3⎡{\log\;}_{2}(KN_{S}/N_{\mathrm{NC}})⎤+⎡\log_{2}\left(N_{\mathrm{NC}}\right)⎤\right]\;\mathrm{bits}, \end{array}$$

where $B_{C}$ and $B_{\mathrm{NC}}$ are the same as previously (Equations (16) and (17)).

#### 3.2.6. Use of Non-Coherent Accumulation with Semi-Parallel Implementation

It is again easy to generalize to the case where $N_{A}$ accumulators tests $N_{A}$ delays in parallel. The processing time is the same as the semi-parallel implementation with coherent integration only. Regarding the amount of memory necessary, the memory-based accumulators require

(19) $$\begin{array}{l} M=N_{A}2N_{P}B_{C}+N_{A}N_{P}B_{\mathrm{NC}}\;\mathrm{bits}\\ =N_{A}N_{P}\left(2B_{C}+B_{\mathrm{NC}}\right)\;\mathrm{bits}\\ =N_{A}N_{P}\left[3B_{R}+3⎡{\log\;}_{2}\left(KN_{S}/N_{\mathrm{NC}}\right)⎤+⎡\log_{2}\left(N_{\mathrm{NC}}\right)⎤\right]\;\mathrm{bits}, \end{array}$$

where $B_{C}$ and $B_{\mathrm{NC}}$ are the same as previously (Equations (16) and (17)).

### 3.3. Acquisition of a Pilot Channel with Assistance

This section presents the serial and semi-parallel implementation when $N_{\mathrm{SCB}}$ delays of the secondary code should be tested instead of $N_{S}$, with $1\leq N_{\mathrm{SCB}}<N_{S}$ (for the case $N_{\mathrm{SCB}}=1$, only the serial implementation is considered). Note that since only the number of delays is reduced, the processing time will be reduced, but the amount of memory will not change.

#### 3.3.1. Coherent Integration Only with Serial Implementation

The implementation is exactly the same as when there is no assistance, shown in Figure 6. The timing diagram is also the same, shown in Figure 7, the only difference is that the part that repeats is repeated $N_{\mathrm{SCB}}$ times instead of $N_{S}$ times. The processing time for one frequency bin is thus,

(20) $$\begin{array}{l} T_{\mathrm{FB}}=KN_{\mathrm{FFT}}N_{S}N_{\mathrm{SCB}}+2\left(N_{\mathrm{FFT}}+L\right)-\left(N_{P}+N_{Z}\right)\\ =KN_{S}N_{\mathrm{SCB}}\left(2N_{P}+N_{Z}\right)+3N_{P}+N_{Z}+2L\\ =N_{P}\left(2KN_{S}N_{\mathrm{SCB}}+3\right)+N_{Z}\left(KN_{S}N_{\mathrm{SCB}}+1\right)+2L. \end{array}$$

Therefore, the processing time is approximately divided by $N_{S}/N_{\mathrm{SCB}}$ compared to the case without assistance, as expected. Note that if the assistance is perfect, i.e., $N_{\mathrm{SCB}}=1$, the processing time becomes the same as the one for the parallel implementation without assistance (Equation (9)), as expected.

#### 3.3.2. Coherent Integration Only with Semi-Parallel Implementation

From the previous cases, it is easy to generalize to the case where $N_{A}$ accumulators test $N_{A}$ delays in parallel among the $N_{\mathrm{SCB}}$ possible. The processing time for one frequency bin is

(21) $$\begin{array}{l} T_{\mathrm{FB}}=KN_{\mathrm{FFT}}N_{S}⎡\frac{N_{\mathrm{SCB}}}{N_{A}}⎤+2\left(N_{\mathrm{FFT}}+L\right)-\left(N_{P}+N_{Z}\right)\\ =KN_{S}⎡\frac{N_{\mathrm{SCB}}}{N_{A}}⎤\left(2N_{P}+N_{Z}\right)+3N_{P}+N_{Z}+2L\\ =N_{P}\left(2KN_{S}⎡\frac{N_{\mathrm{SCB}}}{N_{A}}⎤+3\right)+N_{Z}\left(KN_{S}⎡\frac{N_{\mathrm{SCB}}}{N_{A}}⎤+1\right)+2L. \end{array}$$

#### 3.3.3. Use of Non-Coherent Accumulation with Serial Implementation

The implementation is exactly the same as when there is no assistance, shown in Figure 9. The timing diagram is also the same, shown in Figure 7, the only difference is that the part that repeats is repeated $N_{\mathrm{SCB}}$ times instead of $N_{S}$ times, as in Section 3.3.1. Therefore, the processing time for one frequency bin is given in Equation (20).

#### 3.3.4. Use of Non-Coherent Accumulation with Semi-Parallel Implementation

In the same way, is exactly the same as when there is no assistance, and the processing time is the same as the semi-parallel coherent only implementation—therefore the processing time is given in Equation (21).

### 3.4. Summary

To have a better overview of the previous expressions, Table 2 summarizes the processing time and the memory requirements for the different implementations.

## 4. Application to GPS and Galileo Signals

### 4.1. Determination of the Integration Time

Now, let us compare the different implementations with actual parameters. To have a good overview of the performance, several sensitivities are selected, from −140 dBm to −160 dBm with a step of 5 dBm to cover moderate to high sensitivities. However, as mentioned in the introduction and shown in Table 1, the expected power is not the same for the different signals. Therefore, to perform a fair comparison, the sensitivities should be adapted for each signal, and the values above would be for one signal only. Here the GPS L1 C/A has been chosen as reference. Since the GPS L5 signal has an expected power 1.5 dB above the L1 C/A signal power, the sensitivities considered for the L5 signal are −138.5 dBm, −143.5 dBm, −148.5 dBm, −153.5 dBm, and −158.5 dBm. This allows us to do a fair comparison representative of the reality. The same will apply for the Galileo signals.

**Figure 10:**
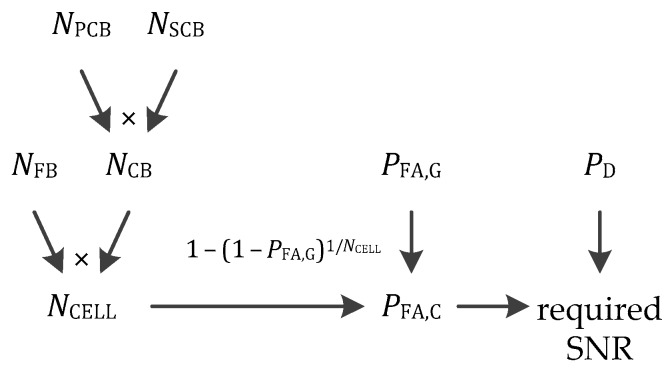
Parameters influencing the Signal-to-Noise Ratio (SNR) required.

Then, again to have a fair comparison, some elements must be considered specifically for each signal: The number of frequency bins and code bins, which influence the size of the search space and consequently influence the required Signal-to-Noise Ratio (SNR), probability of false alarm and probability of detection. This is illustrated in Figure 10, with $N_{\mathrm{FB}}$ the number of frequency bins, $N_{\mathrm{PCB}}$ the number of primary code bins, $N_{\mathrm{SCB}}$ the number of secondary code bins, $N_{\mathrm{CB}}=N_{\mathrm{PCB}}N_{\mathrm{SCB}}$ the number of code bins, $N_{\mathrm{CELL}}=N_{\mathrm{FB}}N_{\mathrm{CB}}$ the number of cells of the search space, $P_{\mathrm{FA},G}$ and $P_{\mathrm{FA},C}$ the global and cell probabilities of false alarm respectively, related by $P_{\mathrm{FA},G}=1-{\left(1-P_{\mathrm{FA},C}\right)}^{N_{\mathrm{CELL}}}$ or $P_{\mathrm{FA},C}=1-{\left(1-P_{\mathrm{FA},C}\right)}^{1/N_{\mathrm{CELL}}}$, and $P_{D}$ the probability of detection [8].

**Figure 11:**
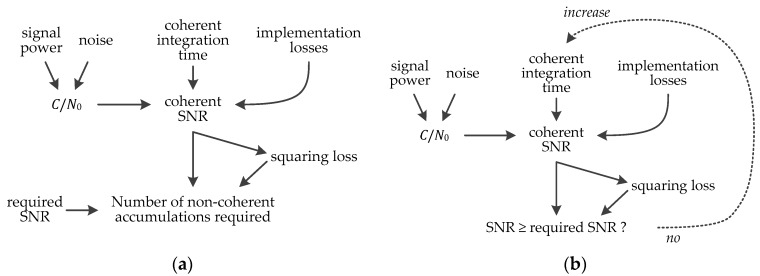
Evaluation of the coherent integration time and number of non-coherent accumulations: (**a**) When the coherent integration time is fixed, and the number of non-coherent accumulations is estimated; (**b**) When there is coherent integration only, and the coherent integration time is estimated.

Finally, once the required SNR is estimated, the coherent integration time and the number of non-coherent accumulations can be evaluated, as shown in Figure 11. The implementation losses depend on the quantization, frequency step, code step, and potential sign transition. Following the outcomes of [32], a 3-bit quantization, a frequency step δf of $2/\left(3T_{C}\right)$, and a code step of 1/2 chip is considered, and since the full bit method and secondary code correlation is used there is no transition loss. Regarding the case of coherent integration only, Figure 11b shows that this is an iterative process. There is even a second iteration not shown in Figure 10 and Figure 11, because increasing the coherent integration will decrease the frequency step (since $\delta f=2/\left(3/T_{C}\right)$). This will increase the number of frequency bins $N_{\mathrm{FB}}$ (since $N_{\mathrm{FB}}=f_{\mathrm{SS}}/\delta f=3f_{\mathrm{SS}}T_{C}/2$ with $f_{\mathrm{SS}}$ the search space), which itself increases the required SNR after Figure 10, which consequently may change the coherent integration time after Figure 11b. Therefore, for a very accurate estimation in the case of coherent integration only, these iterations should be performed.

### 4.2. Comparison of GPS L1 C/A and L5 Signals Acquisition

#### 4.2.1. Determination of Acquisition Parameters

The general parameters independent of the integration time that have been selected are summarized in Table 3 (the spreadsheets used to obtain the further tables are available in supplementary materials). The sampling frequencies are chosen to be as low as possible, because it is desired that the acquisition be as fast as possible, i.e., slightly above twice the chipping rate since these are BPSK signals (it is not exactly twice the chipping rate because the sampling frequency should never be a multiple of the chipping rate, else it would lead to a very bad accuracy in the estimation of the pseudo-ranges and position [33]).

Starting with the L1 C/A signal, a typical search space of ±5 kHz is considered [8], and since the coherent integration time is fixed to 20 ms, the frequency step is $2/\left(3T_{C}\right)=$ 33.3 Hz, implying 2 × 5000/33.3 ≈ 300 frequency bins. The code step is one sample, giving 2048 code bins. The number of cells is thus 300 × 2048 = 614,400. A global probability of false alarm of $10^{-3}$ is chosen to avoid false alarms, which gives $P_{\mathrm{FA},C}=1.63\times 10^{-9}$, and finally a typical probability of detection of 0.9 is chosen following [8], which gives a required final SNR of 17.15 dB.

Since a case of assistance will be considered for the L5 signal, this same assistance should be considered for the L1 C/A signal to make a fair comparison. As mentioned previously, the assistance indicates the current chip of the secondary code (or a small range), and the information needed for this information (time, and receiver and satellites position) can also be used to reduce the frequency search space. Therefore, with assistance, counting an error of 60 Hz due to almanac inaccuracy and an uncertainty of 180 Hz for the receiver velocity (Doppler added by a receiver at 130 km/h in the direction of a satellite) [8], it will be assumed that the search space is reduced to 16 bins, corresponding to ±266.7 Hz.

The same is done for the L5 signal, considering four cases, coherent integration limited to 20 ms or unlimited, and with or without assistance. One difference in the assumptions is that since the L5 frequency is about 75% the one of the L1 frequency, the expected Doppler and the search space for an L5 signal should be reduced. This gives 225 frequency bins without assistance (±3750 Hz), and 12 frequency bins with assistance (±200 Hz). For the L5 signal, the assistance reduces the number of secondary code bin $N_{\mathrm{SCB}}$ and consequently the number of code bins $N_{\mathrm{CB}}$. For the application here, the best assistance is assumed, i.e., the current secondary code chip is known and $N_{\mathrm{SCB}}=1$ and $N_{\mathrm{CB}}=N_{\mathrm{PCB}}$.

Table 4 summarizes all this information. Note that to be very accurate, the number of frequency bins for the unlimited $T_{C}$ case should be higher since $N_{\mathrm{FB}}=3f_{\mathrm{SS}}T_{C}/2$, and an iterative calculation should be performed as mentioned previously. However, this would lead to a different required SNR for each sensitivity, which would be hard to synthesize. Consequently, the same number of frequency bins is considered as simplification, keeping in mind that for these two cases the performance will be a little bit optimistic compared to the real one. Note also that another approximation is made, namely that a Gaussian variable is considered for the detection test. This is the case when the number of non-coherent accumulations is high, however, when there is not or a just few non-coherent accumulations, the variable is not Gaussian, which would affect the way the probabilities of false alarm and detection are calculated [8].

Now that the required SNR is determined, the coherent and total integration times can be estimated following Figure 11, and the values are summarized in Table 5 and Table 6, considering the average and worst case regarding the implementation losses (quantization, frequency step, and code step), namely 0.55 + 0.53 + 1.16 = 2.24 dB and 0.55 + 1.65 + 2.50 = 4.70 dB respectively.

Before going on the evaluation of the different implementations, Table 7 shows the ratio of the total integration time between several cases to quantify the impact of using the L5 signal, unlimited coherent integration time, and assistance. From Table 7, it is clear that the main way to reduce the total integration time is to increase the coherent integration time, especially for very weak signals. Then, the use of L5 brings an interesting reduction, thanks to its higher power, even if the required SNR after correlation is higher due to the bigger search space. Finally, the assistance has the lowest effect on the total integration time, but the reduction is still appreciable (and remember that the assistance reduces the frequency search space, which will have a significant positive impact on the acquisition time).

#### 4.2.2. Determination of Ratio of Complexity for One Frequency Bin

**Figure 12:**
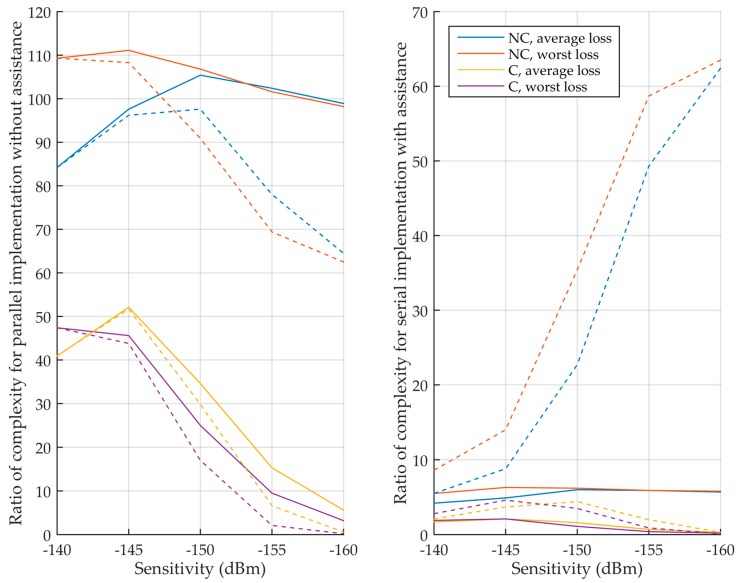
Ratio of complexity (product of processing time ratio and memory ratio) between the L5 and L1 C/A signals. (**Left**) without assistance, (**right**) with assistance. Solid curves do not consider the input memory, while dashed curves consider the input memory.

Using the total integration time of Table 5 and Table 6, the parameters that depend on the integration, i.e., the processing time and the memory requirements, can be determined for the different cases. Table 8 illustrates this in detail for a sensitivity of −150 dBm, where the ratio of processing time and memory between the L5 signal and the L1 C/A signal is given. The final metric of performance is the product of ratios. If nothing is specified, there is no assistance. Then, Table 9 complements it by adding the input memory that stores the input signal. Finally, Table 10 and Figure 12 summarize the product of ratios for all the sensitivities, considering or not the input memory.

First, it can be seen that when the coherent integration time is limited to 20 ms for the L5 signal (non-coherent (NC) case), the L5 signal implementations are much more complex, with products of ratios higher than 100 without assistance.

It can also be noted in Table 8 and Table 9 that without taking into account the input memory, using a parallel, semi-parallel or serial implementation provides about the same performance (except for some semi-parallel that are not efficient, such as the one with nine accumulators because it requires more memory than with seven accumulators for the same processing time since ⎡20/9⎤=⎡20/7⎤=3). When considering the input memory, the parallel implementation becomes more efficient, because such implementation uses a lot of memory, and therefore the input memory represents a smaller portion of it. This is more and more true as the sensitivity decreases. This is why only the parallel implementation is considered in Table 10 and Figure 12 when there is no assistance.

Now, let us focus on the interesting implementations, i.e., when the coherent integration time is not limited. Without assistance, for moderate sensitivities, the L5 implementations are much more complex with products of ratios up to 50. For very high sensitivity, the product of ratios becomes much smaller, even lower than 1 for a sensitivity of −160 dBm. This is mainly because the L1 C/A signal needs a very long integration time, and the input memory can even be larger for the L1 C/A signal than for the L5 signal. With assistance, the products of ratios are much smaller, between 0.1 and 4.6. Therefore, the L5 implementation can be more efficient for the computation of one frequency bin, or if it is less efficient it is by a relatively small ratio.

However, remember that the number of frequency bins for unlimited $T_{C}$ is not the actual one, and it should be much higher with very long coherent integration time (except if the assistance is very accurate, which is possible in some applications [22]).

#### 4.2.3. Determination of Ratio of Complexity for Multiple Frequency Bins

Now that the complexity considering only one frequency bin has been studied, let us consider the real case where it is the acquisition time that matters. For this, the relation between the processing time of one frequency bin and the acquisition time should be determined, i.e., how many frequency bins are searched until the detection of the signal. Of course, this depends on the Doppler frequency of the received signal; however, assuming a uniform distribution of the Doppler frequency over the search space, in average $N_{\mathrm{FB}}/2$ frequency bins are searched. Therefore, to include this in the evaluation of the complexity, the ratio of number of frequency bins searched between the different signals should be estimated, and since the number of frequency bins searched is directly proportional to the total number of bins, the ratio of total number of bins could be used. Two elements will thus be involved: (1) The width of the search space; and (2) the frequency step to browse the search space, which is inversely proportional to coherent integration time. Therefore, compared to the previous results, for the L5 signal with a coherent integration limited to 20 ms (as the L1 C/A signal), it is expected that the final ratio of complexity will be lower since the width of the search space is lower. However, for the L5 signal with longer coherent integration time, the frequency step will decrease, the number of frequency bins will increase and thus the final complexity will increase.

This is shown in Table 11, where the ratio of number of frequency bins is around 0.8 when non-coherent integration is used regardless the assistance, and where the ratio increases with the sensitivity when coherent integration only is used. Applying these ratios to Table 10 gives Table 12 that shows the final complexity ratio, considering the processing time of one frequency bin, the number of frequency bins searched and the memory requirements. It can then be seen that without assistance, the L5 signal is much more complex to acquire. With assistance, the ratios of complexity are lower, but the L5 signal is still more complex to acquire, except for a few cases.

#### 4.2.4. Validation

To validate the previous developments, Matlab simulations have been performed for several sensitivities. GPS L1 C/A and L5 signals have been simulated with a random Doppler frequency within the search space and a random code delay, and acquired following the serial implementations of Section 2. This has been repeated 100 times, and the average of the measurements has been extracted.

**Figure 13:**
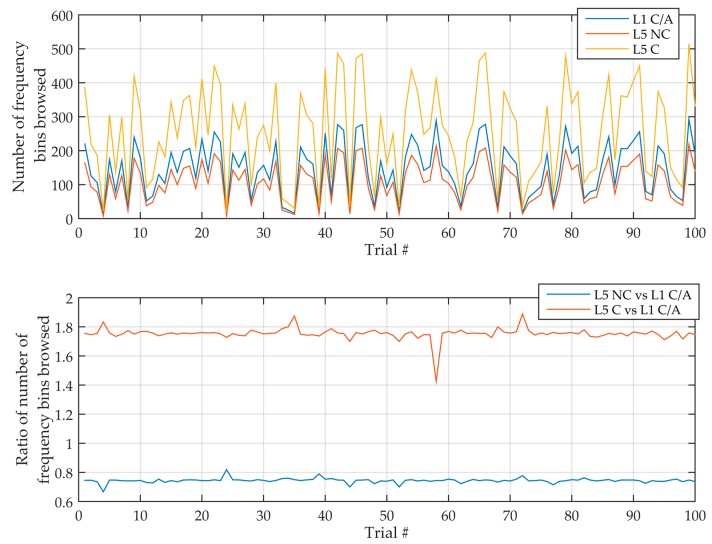
Number of frequency bins browsed (**top**) and ratio (**bottom**) of this over 100 simulations, for a sensitivity of −145 dBm without assistance.

Figure 13 illustrates this for a sensitivity of −145 dBm without assistance, where the ratio measured is relatively constant (the fluctuations are due to the fact that the detection may happen on a bin around the exact one). Then, Table 13 shows the theoretical and measured number of frequency bins and the ratio between L5 and L1. It can be seen that the number of frequency bins searched until detection is around half the total number of frequency bins, as expected. More importantly, it is seen that the measured ratio of the number of frequency bins searched matches the theoretical one, which validates the previous developments.

### 4.3. Comparison of Galileo E1 and E5 Signals Acquisition

The general parameters independent of the integration time that have been selected for Galileo signals are summarized in Table 14. For the Galileo signals, three cases are compared. E1 with a sampling frequency slightly higher 6 times the chipping rate in order to have a code step small enough to give the same losses as in the BPSK case [24]. However, this leads to long sequences (longer than the E5a or E5b). Therefore, E1 with a sampling frequency slightly higher than four times the chipping rate to cover the main lobes of the signals is then considered, but this requires some additional techniques to compensate the correlation shape [34,35]. Finally, for E5a or E5b, a sampling frequency slightly higher than twice the chipping rate is considered, like the L5 signal.

The E1 and E5 secondary codes are both 100 ms long even if the number of chips is different, therefore this value will be considered as coherent integration time when using non-coherent integration.

Starting with the E1 signal, to be fair compared to the parameters selected for the GPS signals, a typical search space of ±4 kHz is considered (since the Galileo satellites have a lower speed and a maximum range rate around 20% lower); and since the coherent integration time is fixed to 100 ms, the frequency step is $2/\left(3T_{C}\right)=$ 6.67 Hz, implying 2 × 4000/6.67 ≈ 1200 frequency bins. The code step is one sample, giving 16,384 primary code bins for a sampling frequency of 4.096 MHz and 24,576 primary code bins for a sampling frequency of 6.144 MHz. Since the E1 secondary code is 25 chips long, the number of cells is 1200 × 16,384 × 25 = 491,520,000 and 1200 × 24,576 × 25 = 737,280,000 respectively. A global probability of false alarm of $10^{-3}$ gives $P_{\mathrm{FA},C}=2.04\times 10^{-12}$ and $P_{\mathrm{FA},C}=1.36\times 10^{-12}$ respectively, and finally a typical probability of detection of 0.9 gives a required final SNR of 18.30 dB and 18.35 dB respectively.

Then, as for the GPS, a case with assistance is also considered, where the current chip of the secondary code is known, and where the frequency search space is reduced to 213.3 Hz (80% of the 266.7 Hz of the GPS L1 C/A), giving 32 frequency bins.

The same is done for the E5 signal, considering four cases, coherent integration limited to 100 ms or unlimited, and with or without assistance. As for GPS, the only difference in the assumptions is that the frequency search space is about 75% the one of the E1 signal, which gives 900 frequency bins without assistance (±3000 Hz), and 24 frequency bins with assistance (±160 Hz).

Table 15 summarizes all this information. The values for the unlimited $T_{C}$ case are considered the same, although the number of frequency bins should be adapted, as already mentioned in Section 4.2.

Next, the coherent and total integration times can be estimated following Figure 11, and the values are summarized in Table 16 and Table 17, considering the average and worst case regarding the implementation losses (same losses as for GPS signals). It can be seen that the integration times are very similar for the both sampling frequencies with the E1 signals. However, the integration time with the E5 signal is much lower in general, thanks to its power that is 2 dB higher.

Then, Table 18 then shows the ratio of the total integration time between several cases to quantify the impact of using unlimited coherent integration time and assistance with Galileo signals. It confirms the outcomes of Table 7 that the best way to reduce the total integration time is to increase the coherent integration time, especially for weak signals, but that having assistance still helps to reduce the integration time in a non-negligible way.

**Figure 14:**
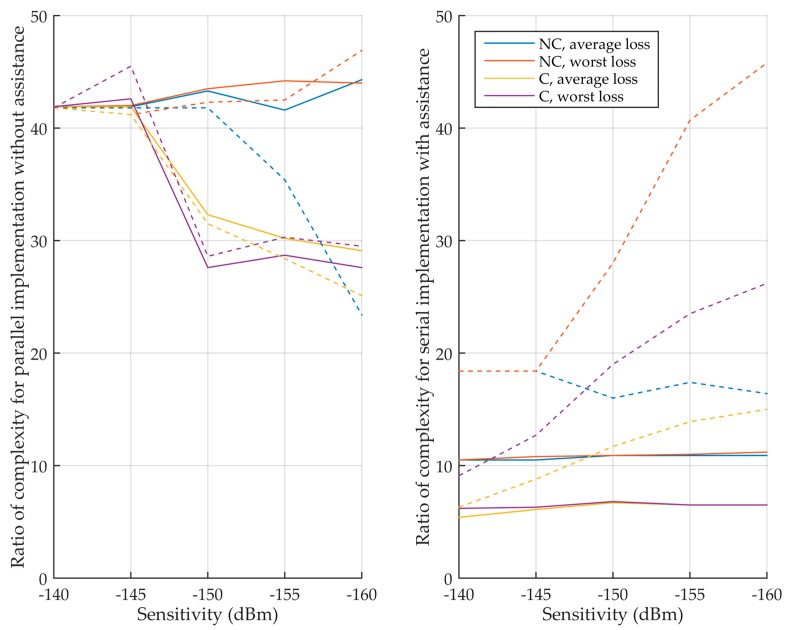
Ratio of complexity (product of processing time ratio and memory ratio) between the E5 signal and the E1 signal with a sampling frequency of 4.096 MHz. (**Left**) without assistance, (**right**) with assistance. Solid curves do not consider the input memory, while dashed curves consider the input memory.

Then, Table 19 and Table 20 and Figure 14 summarize the product of ratios for all the sensitivities, both taking into account the input memory and not consider this factor—comparing E1 with itself for different sampling frequencies, and comparing E5 with E1, respectively.

Table 19 shows that using a sampling frequency of 6.144 MHz instead of 4.096 MHz for the E1 signal triples the complexity most of the time. Therefore, it can be concluded it is certainly more efficient to use a sampling frequency of 4.096 MHz and a technique that modifies the local code to get rid of the side peak [34,35], rather than using a sampling frequency of 6.144 MHz.

Then, Table 20 and Figure 14 show that when there is no assistance, the E5 acquisition is approximately 25 to 45 times more complex. This is due to the very long secondary code to synchronize with (100 chips), which dramatically impacts the complexity.

When there is assistance, without considering the input memory, the E5 acquisition is still more complex, but by a lower ratio, around 6 for coherent only implementation and around 11 when non-coherent accumulation is used. One may note that if the ratio between E5 and E1 with a sampling frequency of 6.144 MHz was calculated (by simply diving the ratio of Table 20 by those of Table 19), the E5 could be only twice more complex than E1 for coherent only implementation. This is impressive, noting that the sampling frequency of the E5 is more than three times higher and the primary code is more than twice longer.

Finally, still with assistance, if the input memory is considered, the complexity increases and increases with the sensitivity, because at this time the sampling frequency of 20.48 MHz of the E5 signal becomes a huge burden.

Now, we can take into account the average number of frequency bins searched until detection to obtain the final ratios of complexity. Table 21 shows the ratio of number of frequency bins between the signals. As for the GPS, when there is non-coherent integration and that the coherent integration time is the same for both signals, the ratio depends only on the width of the search space, hence the ratio of 0.8. Then, with coherent integration only, since both signals are not limited in the coherent integration time (unlike the comparison of GPS L1 C/A and L5), the ratio is in favor of the E5 signal because it requires a smaller integration time thanks to its higher power.

Finally, applying these ratios to Table 20 gives Table 22 that shows the final complexity ratio, and it can be seen that even if the ratios are lower than Table 20, the E5 signal is still more complex than the E1.

## 5. Conclusions

This paper performed a very detailed comparison of the complexity of the acquisition of L1 and L5 bands signals, to determine which signal should be acquired first to then help the other, to verify the common thought that L5 band signals acquisition is more complex, and especially to quantify this ratio.

Such detailed comparison is needed because many parameters influence positively or negatively each band and each signal, such as the chipping rate, the sampling frequency, the carrier frequency, the length of the primary and secondary codes, the signal power, or the availability of a pilot channel; it is therefore difficult to make accurate estimation.

In a first part, general expressions of the processing time and memory requirements have been presented and are summarized in Table 2. Such expressions are applicable for any GNSS signal, and depend on several parameters. Some parameters depend on the signal considered, such as the number of samples in one code period (which depends on the code length and sampling frequency), or the length of the secondary code; and some parameters do not depend on the signal considered but depend on the context and design, such as the number of non-coherent integrations or the number of bits used for the quantization.

In a second part, the comparisons have been performed by evaluating the aforementioned expressions. An accurate estimation of the processing time and memory requirements has thus been done, providing all the details of the methodology, and considering many details (such as the search space that influences the probability of false alarm at the cell level and the signal-to-noise ratio required). In order to have a general view and not just one example, the following has been considered: Five sensitivities (from −140 dBm to −160 dBm with a step of 5 dBm); both unassisted and assisted case; and both coherent only integration (when applicable) and the use of non-coherent integration. Studies have been included to evaluate the impact of each element (Table 7 and Table 18).

For GPS, the L1 C/A and L5 signals have been compared. Table 10 and Figure 12 provide the ratio between the complexity of each of them considering the processing time of one frequency bin and the memory requirements, and Table 12 provides the final ratio of complexity which takes also into account the average number of frequency bins browsed until the signal detection. The developments have been validated by Matlab simulations (Table 13). The acquisition of the L5 signal is most of the time more complex. Without assistance, if the sensitivity or the coherent integration time is moderate, acquiring the L5 signal is much more complex (ratios higher than 50 most often, i.e., it may require 50 times more memory, or have a processing time that is 50 times longer, or e.g., 10 times more memory, with a 5 times longer processing time). For very high sensitivity with long coherent integration time, the complexity ratio for one frequency bin is smaller, but still to the detriment of the L5 signal, except at −160 dBm. With assistance, the complexity ratios are smaller for any sensitivity, but only those for unlimited coherent integration time are of interests, since the ratios for one frequency bin become between 0.1 and 4.6. i.e., the GPS L5 signal acquisition may be significantly less complex than the GPS L1 C/A signal one in some high sensitivity cases if only one or few frequency bins that have to be tested (which would require very accurate Doppler assistance). When considering more frequency bins, the L5 signal is more complex to acquire, except few cases.

For Galileo, the E1 signal has been compared with itself first, considering two different sampling frequencies, 4.096 MHz and 6.144 MHz, the first one being close to the minimum and the second one offering the same code loss as a BPSK signals sampled at about twice the chipping rate. Table 19 provides the ratios of complexity, and shows using a sampling frequency of 6.144 triples the complexity most of the time. It is therefore recommended to use the minimum sampling frequency of 4.096 MHz and use techniques to remove the side peaks of the main correlation peak.

Then, the E1 and E5 signals have been compared. Table 20 and Figure 14 provide the ratios of complexity for one frequency bin, and shows that the acquisition of the E5 signal is always more complex. Without assistance, acquiring the E5 signal is much more complex (ratios higher between 25 and 45). With assistance, the complexity ratios are smaller for any sensitivity, the lowest ratios being for unlimited coherent integration times; but the minimum ratio is still around 6 (without considering the input memory, else the ratios increase with the sensitivity), which makes the Galileo E5 signal acquisition significantly more complex than the Galileo E1 one even in the best case. Considering more frequency bins reduces these ratios (Table 22), however the E5 signal is still more complex to acquire.

In conclusion, the GPS and Galileo L5 band signals are overall more complex to acquire than the GPS and Galileo L1 band signals, although in some particular cases the difference may be negligible or limited. In particular, the L5 signal could show better performance in presence of very accurate assistance to avoid a significant increase of the number of frequency bins when using very long coherent integration times.

The methodology and the expressions and developments provided in this paper can be easily used to compare the complexity of current or future GNSS signals in specific cases for a wide variety of hardware implementations.

## Figures and Tables

**Table 1:** Summary of the characteristics of the considered Global Navigation Satellite System (GNSS) signals. BPSK stand for Binary Phase Shift Keying, CBOC stands for Code Binary Offset Carrier.

System		L1 C/A	L5		E1		E5a		E5b	
Center frequency (MHz)		1575.42	1176.45		1575.42		1176.45		1176.45	
Channel combination		-	Quadrature		Code multiplexing		Quadrature		Quadrature	
Channel		Data	Data	Pilot	Data	Pilot	Data	Pilot	Data	Pilot
Bandwidth		2.046	20.46	20.46	14.322	14.322	20.46	20.46	20.46	20.46
Minimum bandwidth		2.046	20.46	20.46	4.092	4.092	20.46	20.46	20.46	20.46
Modulation		BPSK (1)	BPSK (10)	BPSK (10)	CBOC (6,1)		BPSK (10)	BPSK (10)	BPSK (10)	BPSK (10)
Sub-carrier frequency (MHz)		-	-	-	1.023 & 6.138	1.023 & 6.138	-	-	-	-
Primary code	Chipping rate (Mchip/s)	1.023	10.23	10.23	1.023	1.023	10.23	10.23	10.23	10.23
	Length (chip)	1023	10,230	10,230	4092	4092	10,230	10,230	10,230	10,230
	Length (ms)	1	1	1	4	4	1	1	1	1
Secondary code	Chipping rate (chip/s)	-	1000	1000	-	250	1000	1000	1000	1000
	Length (chip)	-	10	20	-	25	20	100	4	100
	Length (ms)	-	10	20	-	100	20	100	4	100
Data rate	(symbol/s)	50	100	-	250	-	50	-	250	-
	(bit/s)	50	50	-	125	-	25	-	125	-
Minimum power received on Earth	(dBW)	−158.5	−157	−157	−160	−160	−158	−158	−158	−158
	(dBm)	−128.5	−127	−127	−130	−130	−128	−128	−128	−128

**Table 2:** Summary of the processing time and memory requirements of the different implementations.

Implementation		Processing Time of One Frequency Bin $T_{FB}$ (Clock Cycle)	Memory (bit)	Figure
Data	Parallel NC	$N_{P}\left[\left(K+1\right)2N_{D}+1\right]+N_{Z}\left[\left(K+1\right)N_{D}\right]+2L$	$N_{D}N_{P}\left[3B_{R}+3⎡\log_{2}\left(N_{D}\right)⎤+⎡\log_{2}\left(K\right)⎤\right]$	2, 3
Pilot	Parallel C	$N_{P}\left(2KN_{S}+3\right)+N_{Z}\left(KN_{S}+1\right)+2L$	$N_{S}2N_{P}\left(B_{R}+⎡\log_{2}\left(KN_{S}\right)⎤\right)$	4, 5
	Parallel NC	$N_{P}\left(2KN_{S}+3\right)+N_{Z}\left(KN_{S}+1\right)+2L$	$N_{S}N_{P}\left[3B_{R}+3⎡\log_{2}\left(KN_{S}/N_{\mathrm{NC}}\right)⎤+⎡\log_{2}\left(N_{\mathrm{NC}}\right)⎤\right]$	5, 8
	Serial C	$N_{P}\left(2KN_{S}^{2}+3\right)+N_{Z}\left(KN_{S}^{2}+1\right)+2L$	$2N_{P}\left(B_{R}+⎡\log_{2}\left(KN_{S}\right)⎤\right)$	6, 7 *
	Serial C with assistance	$N_{P}\left(2KN_{S}N_{\mathrm{SCB}}+3\right)+N_{Z}\left(KN_{S}N_{\mathrm{SCB}}+1\right)+2L$	$2N_{P}\left(B_{R}+⎡\log_{2}\left(KN_{S}\right)⎤\right)$	6, 7 *
	Serial NC	$N_{P}\left(2KN_{S}^{2}+3\right)+N_{Z}\left(KN_{S}^{2}+1\right)+2L$	$N_{P}\left[3B_{R}+3⎡\log_{2}\left(KN_{S}/N_{\mathrm{NC}}\right)⎤+⎡\log_{2}\left(N_{\mathrm{NC}}\right)⎤\right]$	7 *, 9
	Serial NC with assistance	$N_{P}\left(2KN_{S}N_{\mathrm{SCB}}+3\right)+N_{Z}\left(KN_{S}N_{\mathrm{SCB}}+1\right)+2L$	$N_{P}\left[3B_{R}+3⎡\log_{2}\left(KN_{S}/N_{\mathrm{NC}}\right)⎤+⎡\log_{2}\left(N_{\mathrm{NC}}\right)⎤\right]$	7 *, 9
	Semi-parallel C	$N_{P}\left(2KN_{S}⎡\frac{N_{S}}{N_{A}}⎤+3\right)+N_{Z}\left(KN_{S}⎡\frac{N_{S}}{N_{A}}⎤+1\right)+2L$	$N_{A}2N_{P}\left(B_{R}+⎡\log_{2}\left(KN_{S}\right)⎤\right)$	
	Semi-parallel C with assistance	$\begin{array}{l} N_{P}\left(2KN_{S}⎡\frac{N_{S}N_{\mathrm{SCB}}}{N_{A}}⎤+3\right)\\ +N_{Z}\left(KN_{S}⎡\frac{N_{S}N_{\mathrm{SCB}}}{N_{A}}⎤+1\right)+2L \end{array}$	$N_{A}2N_{P}\left(B_{R}+⎡\log_{2}(KN_{S})⎤\right)$	
	Semi-parallel NC	$N_{P}\left(2KN_{S}⎡\frac{N_{S}}{N_{A}}⎤+3\right)+N_{Z}\left(KN_{S}⎡\frac{N_{S}}{N_{A}}⎤+1\right)+2L$	$N_{A}N_{P}\left[3B_{R}+3⎡\log_{2}\left(KN_{S}/N_{\mathrm{NC}}\right)⎤+⎡\log_{2}\left(N_{\mathrm{NC}}\right)⎤\right]$	
	Semi-parallel NC with assistance	$\begin{array}{l} N_{P}\left(2KN_{S}⎡\frac{N_{S}N_{\mathrm{SCB}}}{N_{A}}⎤+3\right)\\ +N_{Z}\left(KN_{S}⎡\frac{N_{S}N_{\mathrm{SCB}}}{N_{A}}⎤+1\right)+2L \end{array}$	$N_{A}N_{P}\left[3B_{R}+3⎡\log_{2}\left(KN_{S}/N_{\mathrm{NC}}\right)⎤+⎡\log_{2}\left(N_{\mathrm{NC}}\right)⎤\right]$	

* not exactly the same, but similar.

**Table 3:** Parameters independent of the integration time selected for Global Positioning System (GPS) signals.

Signal	$f_{S}$	$N_{P}$	$N_{\mathrm{FFT}}$	$N_{Z}$	$N_{D}$	$N_{S}$	$B_{R}$
L1 C/A	2.048 MHz	2048	4096	0	20	-	16
L5	20.48 MHz	20,480	65,536	24,576	-	20	16

**Table 4:** Search space and statistical parameters for $P_{\mathrm{FA},G}=10^{-3}$ and $P_{D}=0.9$ for different contexts with GPS signals.

Context	Signal	$T_{C}$	$N_{\mathrm{FB}}$	$N_{\mathrm{CB}}$	$N_{\mathrm{CELL}}$	$P_{\mathrm{FA},C}$	SNR
no assistance	L1 C/A	20 ms	300	2048	614,400	1.63 × 10^−9^	17.15 dB
	L5	20 ms	225	409,600	92,160,000	1.09 × 10^−11^	18.04 dB
	L5	unlimited	225	409,600	92,160,000	1.09 × 10^−11^	18.04 dB
assistance	L1 C/A	20 ms	16	2048	32,768	3.05 × 10^−8^	16.52 dB
	L5	20 ms	12	20,480	245,760	4.07 × 10^−9^	16.96 dB
	L5	unlimited	12	20,480	245,760	4.07 × 10^−9^	16.96 dB

**Table 5:** Total integration time $T_{\mathrm{IT}}=T_{C}\times N_{\mathrm{NC}}$ as function of the sensitivity and context with GPS signals, for average losses. Note that the sensitivities given are for the L1 C/A signal; those of L5 are 1.5 dB higher.

Context	Signal	$T_{C}$	−140 dBm	−145 dBm	−150 dBm	−155 dBm	−160 dBm
no assistance	L1 C/A	20 ms	20 × 1 = 20	20 × 4 = 80	20 × 23 = 460	20 × 170 = 3400	20 × 1536 = 30,720
	L5	20 ms	20 × 1 = 20	20 × 3 = 60	20 × 16 = 320	20 × 111 = 2220	20 × 963 = 19,260
	L5	unlimited	15 × 1 = 15	47 × 1 = 47	146 × 1 = 146	462 × 1 = 462	1460 × 1 = 1460
assistance	L1 C/A	20 ms	20 × 1 = 20	20 × 4 = 80	20 × 20 = 400	20 × 147 = 2940	20 × 1328 = 26,560
	L5	20 ms	20 × 1 = 20	20 × 3 = 60	20 × 13 = 260	20 × 86 = 1720	20 × 751 = 15,020
	L5	unlimited	12 × 1 = 12	38 × 1 = 38	120 × 1 = 120	377 × 1 = 377	1191 × 1 = 1191

**Table 6:** Total integration time $T_{\mathrm{IT}}=T_{C}\times N_{\mathrm{NC}}$ as function of the sensitivity and context with GPS signals, for worst losses. Note that the sensitivities given are for the L1 C/A signal; those of L5 are 1.5 dB higher.

Context	Signal	$T_{C}$	−140 dBm	−145 dBm	−150 dBm	−155 dBm	−160 dBm
no assistance	L1 C/A	20 ms	20 × 2 = 40	20 × 9 = 180	20 × 59 = 1180	20 × 495 = 9900	20 × 4670 = 93,400
	L5	20 ms	20 × 2 = 40	20 × 7 = 140	20 × 40 = 800	20 × 315 = 6300	20 × 2906 = 58,120
	L5	unlimited	26 × 1 = 26	82 × 1 = 82	258 × 1 = 258	814 × 1 = 814	2573 × 1 = 2573
assistance	L1 C/A	20 ms	20 × 2 = 40	20 × 8 = 160	20 × 51 = 1020	20 × 428 = 8560	20 × 4040 = 80,800
	L5	20 ms	20 × 2 = 40	20 × 5 = 100	20 × 31 = 620	20 × 246 = 4920	20 × 2266 = 45,320
	L5	unlimited	21 × 1 = 21	67 × 1 = 67	210 × 1 = 210	664 × 1 = 664	2099 × 1 = 2099

**Table 7:** Ratio of total integration time (%) for different comparisons with GPS signals. Top value is for average losses, and bottom value is for worst losses. Note that the sensitivities given are for the L1 C/A signal; those of L5 are 1.5 dB higher.

Comparison	Fixed Element	−140 dBm	−145 dBm	−150 dBm	−155 dBm	−160 dBm
Impact of using L5: L5 verses L1 C/A with $T_{C}=20$ ms	no assistance	100	75	70	65	63
		100	78	68	64	62
	assistance	100	65	65	59	57
		100	61	61	57	56
Impact of $T_{C}$: Unlimited verses 20 ms with L5 signal	no assistance	75	78	46	21	8
		65	59	32	13	4
	assistance	60	63	46	22	8
		53	67	34	13	5
Impact of assistance: Assistance verses no assistance	L1 signal	100	100	87	86	86
		100	89	86	86	87
	L5 signal ($T_{C}=20$ ms)	100	100	81	77	78
		100	71	78	78	78
	L5 signal ($T_{C}$ unlimited)	80	81	82	82	82
		81	82	81	82	82

**Table 8:** Processing time (clock cycle) and memory requirements (bit) of the different implementations for a sensitivity of −150 dBm with average losses. Ratios are computed with the values of the L1 C/A signal.

Implementation	K	$N_{\mathrm{NC}}$	$N_{A}$	$B_{C}$	$B_{\mathrm{NC}}$	$T_{\mathrm{FB}}$	Ratio of $T_{\mathrm{FB}}$	M	Ratio of M	Product of Ratios
L1 C/A Parallel NC	23	23	20	21	26	1,968,128		2,785,280		
L5 Parallel NC	16	16	20	21	25	21,057,536	10.7	27,443,200	9.9	105.4
L5 Semi-parallel NC	16	16	10	21	25	42,029,056	21.4	13,751,600	4.9	105.2
L5 Semi-parallel NC	16	16	9	21	25	63,000,576	32.0	12,349,440	4.4	141.9
L5 Semi-parallel NC	16	16	8	21	25	63,000,576	32.0	10,977,280	3.9	126.2
L5 Semi-parallel NC	16	16	7	21	25	63,000,576	32.0	9,605,120	3.4	110.4
L5 Semi-parallel NC	16	16	6	21	25	83,972,096	42.7	8,232,960	3.0	126.1
L5 Semi-parallel NC	16	16	5	21	25	83,972,096	42.7	6,860,800	2.5	105.1
L5 Semi-parallel NC	16	16	4	21	25	104,943,616	53.3	5,488,640	2.0	105.1
L5 Semi-parallel NC	16	16	3	21	25	146,886,656	74.6	4,116,480	1.5	110.3
L5 Semi-parallel NC	16	16	2	21	25	209,801,216	106.6	2,744,320	1.0	105.0
L5 Serial NC	16	16	1	21	25	419,516,416	213.2	1,372,160	0.5	105.0
L5 Parallel C	7.30	1	20	24	-	9,654,272	4.9	19,660,800	7.1	34.6
L5 Semi-parallel C	7.30	1	10	24	-	19,222,528	9.8	9,830,400	3.5	34.5
L5 Semi-parallel C	7.30	1	9	24	-	28,790,784	14.6	8,847,360	3.2	46.5
L5 Semi-parallel C	7.30	1	8	24	-	28,790,784	14.6	7,864,320	2.8	41.3
L5 Semi-parallel C	7.30	1	7	24	-	28,790,784	14.6	6,881,280	2.5	36.1
L5 Semi-parallel C	7.30	1	6	24	-	38,359,040	19.5	5,898,240	2.1	41.3
L5 Semi-parallel C	7.30	1	5	24	-	38,359,040	19.5	4,915,200	1.8	34.4
L5 Semi-parallel C	7.30	1	4	24	-	47,927,296	24.4	3,932,160	1.4	34.4
L5 Semi-parallel C	7.30	1	3	24	-	67,063,808	34.1	2,949,120	1.1	36.1
L5 Semi-parallel C	7.30	1	2	24	-	95,768,576	48.7	1,966,080	0.7	34.3
L5 Serial C	7.30	1	1	24	-	191,451,136	97.3	983,040	0.4	34.3
L1 C/A Parallel + assist.	20	20	20	21	26	1,722,368		2,785,280	1.0	
L5 Serial NC + assist.	13	13	1	21	25	21,057,536	12.2	1,372,160	0.5	6.0
L5 Serial C + assist.	6.00	1	1	23	-	7,950,336	4.6	942,080	0.3	1.6

**Table 9:** Memory requirements (bit) of the different implementations for a sensitivity of −150 dBm with average losses, considering the input memory. Ratios are computed with the values of the L1 C/A signal.

Implementation	$T_{\mathrm{IT}}$	Input Memory (bit)	Total Memory (bit)	Memory Ratio	Product of Ratios
L1 C/A Parallel NC	460	942,080	3,727,360		
L5 Parallel NC	320	6,553,600	33,996,800	9.1	97.6
L5 Semi-parallel NC	320	6,553,600	20,275,200	5.4	116.2
L5 Semi-parallel NC	320	6,553,600	18,903,040	5.1	162.3
L5 Semi-parallel NC	320	6,553,600	17,530,880	4.7	150.6
L5 Semi-parallel NC	320	6,553,600	16,158,720	4.3	138.8
L5 Semi-parallel NC	320	6,553,600	14,786,560	4.0	169.3
L5 Semi-parallel NC	320	6,553,600	13,414,400	3.6	153.6
L5 Semi-parallel NC	320	6,553,600	12,042,240	3.2	172.3
L5 Semi-parallel NC	320	6,553,600	10,670,080	2.9	213.6
L5 Semi-parallel NC	320	6,553,600	9,297,920	2.5	265.9
L5 Serial NC	320	6,553,600	7,925,760	2.1	453.2
L5 Parallel C	146	2,990,080	22,650,880	6.1	29.8
L5 Semi-parallel C	146	2,990,080	12,820,480	3.4	33.6
L5 Semi-parallel C	146	2,990,080	11,837,440	3.2	46.5
L5 Semi-parallel C	146	2,990,080	10,854,400	2.9	42.6
L5 Semi-parallel C	146	2,990,080	9,871,360	2.6	38.7
L5 Semi-parallel C	146	2,990,080	8,888,320	2.4	46.5
L5 Semi-parallel C	146	2,990,080	7,905,280	2.1	41.3
L5 Semi-parallel C	146	2,990,080	6,922,240	1.9	45.2
L5 Semi-parallel C	146	2,990,080	5,939,200	1.6	54.3
L5 Semi-parallel C	146	2,990,080	4,956,160	1.3	64.7
L5 Serial C	146	2,990,080	3,973,120	1.1	103.7
L1 C/A Parallel + assist.	400	819,200	3,604,480		
L5 Serial NC + assist.	260	5,324,800	6,696,960	1.9	22.7
L5 Serial C + assist.	120	2,457,600	3,399,680	0.9	4.4

**Table 10:** Ratio of complexity (product of processing time ratio and memory ratio) between the L5 and L1 C/A signals. Top rows do not consider the input memory, while bottom rows consider the input memory.

Sensitivity	Parallel without Assistance				Serial with Assistance			
	Non-Coherent		Coherent Only		Non-Coherent		Coherent Only	
	Average	Worst	Average	Worst	Average	Worst	Average	Worst
−140 dBm	84.2	109.3	40.9	47.4	4.2	5.5	1.7	1.9
−145 dBm	97.6	111.1	52.1	45.6	4.9	6.3	2.1	2.1
−150 dBm	105.4	106.8	34.6	25.0	6.0	6.2	1.6	1.1
−155 dBm	102.4	101.6	15.3	9.5	5.9	5.9	0.7	0.4
−160 dBm	98.9	98.2	5.6	3.2	5.7	5.8	0.3	0.2
−140 dBm	84.2	109.3	41.0	47.4	5.5	8.6	2.1	2.8
−145 dBm	96.2	108.3	51.7	43.8	8.8	14.0	3.7	4.6
−150 dBm	97.6	90.9	29.8	17.0	22.7	35.4	4.4	3.5
−155 dBm	78.0	69.4	6.6	2.1	49.3	58.7	2.0	0.9
−160 dBm	64.5	62.5	0.6	0.2	62.4	63.5	0.3	0.1

**Table 11:** Ratio of number of frequency bins between the L5 and L1 C/A signals.

Sensitivity	Parallel without Assistance				Serial with Assistance			
	Non-Coherent		Coherent Only		Non-Coherent		Coherent Only	
	Average	Worst	Average	Worst	Average	Worst	Average	Worst
−140 dBm	0.8	0.8	0.6	1.0	0.8	0.8	0.5	0.8
−145 dBm	0.8	0.8	1.8	3.1	0.8	0.8	1.4	2.5
−150 dBm	0.8	0.8	5.5	9.7	0.8	0.8	4.5	7.9
−155 dBm	0.8	0.8	17.3	30.5	0.8	0.8	14.1	24.9
−160 dBm	0.8	0.8	54.8	96.5	0.8	0.8	44.7	78.7

**Table 12:** Final ratio of complexity (product of processing time ratio, memory ratio and frequency bins ratio) between the L5 and L1 C/A signals obtained as the product of Table 10 and Table 11. Top rows do not consider the input memory, while bottom rows consider the input memory.

Sensitivity	Parallel without Assistance				Serial with Assistance			
	Non-Coherent		Coherent Only		Non-Coherent		Coherent Only	
	Average	Worst	Average	Worst	Average	Worst	Average	Worst
−140 dBm	63.1	81.9	23.0	46.2	3.2	4.1	0.8	1.5
−145 dBm	73.2	83.3	91.8	140.4	3.7	4.7	3.0	5.3
−150 dBm	79.1	80.1	189.6	242.3	4.5	4.6	7.0	8.9
−155 dBm	76.8	76.2	264.4	289.9	4.4	4.4	10.2	11.2
−160 dBm	74.2	73.7	303.9	313.5	4.3	4.3	11.7	12.2
−140 dBm	63.1	81.9	23.1	46.2	4.1	6.5	1.0	2.2
−145 dBm	72.1	81.2	91.1	134.8	6.6	10.5	5.3	11.6
−150 dBm	73.2	68.2	163.2	164.3	17.0	26.5	19.6	27.5
−155 dBm	58.5	52.0	113.9	65.6	37.0	44.0	28.3	22.1
−160 dBm	48.4	46.9	32.8	16.6	46.8	47.6	14.2	8.6

**Table 13:** Theoretical and average of the measured number of frequency bins and their ratio between L5 and L1.

	Signals	No Assistance			Assistance		
		−140 dBm	−145 dBm	−150 dBm	−140 dBm	−145 dBm	−150 dBm
Number of frequency bins in the search space	L1 C/A	301	301	301	17	17	17
	L5 NC	225	225	225	13	13	13
	L5 C	169	529	1643	7	23	73
Number of frequency bins searched until detection	L1 C/A	135.5	146.9	139.9	7.3	7.0	7.9
	L5 NC	100.5	109.4	104.3	5.1	4.9	5.6
	L5 C	75.4	256.6	711.0	3.2	10.1	35.7
Ratio of number of frequency bin in the search space	L5 NC verses L1 C/A	0.75	0.75	0.75	0.76	0.76	0.76
	L5 C verses L1 C/A	0.56	1.76	5.46	0.41	1.35	4.29
Ratio of number of frequency bins searched until detection	L5 NC verses L1 C/A	0.74	0.74	0.75	0.70	0.71	0.71
	L5 C verses L1 C/A	0.56	1.75	5.08	0.43	1.44	4.52

**Table 14:** Parameters independent of the integration time selected for Galileo signals.

Signal	$f_{S}$	$N_{P}$	$N_{\mathrm{FFT}}$	$N_{Z}$	$N_{D}$	$N_{S}$	$B_{R}$
E1	4.096 MHz	16,384	32,768	0	-	25	16
E1	6.144 MHz	24,576	65,536	16,384	-	25	16
E5a	20.48 MHz	20,480	65,536	24,576	-	100	16

**Table 15:** Search space and statistical parameters for $P_{\mathrm{FA},G}=10^{-3}$ and $P_{D}=0.9$ for different contexts with Galileo signals. The same values are considered for unlimited $T_{C}$.

Context	Signal	$T_{C}$	$N_{\mathrm{FB}}$	$N_{\mathrm{CB}}$	$N_{\mathrm{CELL}}$	$P_{\mathrm{FA},C}$	SNR
no assistance	E1 (4.096)	100 ms	1200	409,600	491,520,000	2.04 × 10^−12^	18.30 dB
	E1 (6.144)	100 ms	1200	614,400	737,280,000	1.36 × 10^−12^	18.35 dB
	E5	100 ms	900	2,048,000	1,843,200,000	5.40 × 10^−13^	18.49 dB
assistance	E1 (4.096)	100 ms	32	16,384	524,288	1.91 × 10^−9^	17.12 dB
	E1 (6.144)	100 ms	32	24,576	786,432	1.27 × 10^−9^	17.20 dB
	E5	100 ms	24	20,480	491,520	2.04 × 10^−9^	17.10 dB

**Table 16:** Total integration time $T_{\mathrm{IT}}=T_{C}\times N_{\mathrm{NC}}$ as function of the sensitivity and context with Galileo signals, for average losses. Note that the sensitivities given are for the L1 C/A signal; those of E1 are 1.5 dB lower and those of E5 are 0.5 dB higher.

Context	Signal	$T_{C}$	−140 dBm	−145 dBm	−150 dBm	−155 dBm	−160 dBm
no assistance	E1 (4.096)	100 ms	100 × 1 = 100	100 × 1 = 100	100 × 5 = 500	100 × 24 = 2400	100 × 179 = 17,900
	E1 (6.144)	100 ms	100 × 1 = 100	100 × 1 = 100	100 × 5 = 500	100 × 25 = 2500	100 × 182 = 18,200
	E5	100 ms	100 × 1 = 100	100 × 1 = 100	100 × 3 = 300	100 × 13 = 1300	100 × 82 = 8200
	E1 (4.096)	unlimited	100 × 1 = 100	100 × 1 = 100	400 × 1 = 400	1000 × 1 = 1000	3100 × 1 = 3100
	E1 (6.144)	unlimited	100 × 1 = 100	100 × 1 = 100	400 × 1 = 400	1000 × 1 = 1000	3100 × 1 = 3100
	E5	unlimited	100 × 1 = 100	100 × 1 = 100	300 × 1 = 300	700 × 1 = 700	2100 × 1 = 2100
assistance	E1 (4.096)	100 ms	100 × 1 = 100	100 × 1 = 100	100 × 4 = 400	100 × 19 = 1900	100 × 137 = 13,700
	E1 (6.144)	100 ms	100 × 1 = 100	100 × 1 = 100	100 × 4 = 400	100 × 19 = 1900	100 × 139 = 13,900
	E5	100 ms	100 × 1 = 100	100 × 1 = 100	100 × 2 = 200	100 × 9 = 900	100 × 59 = 5900
	E1 (4.096)	unlimited	28 × 1 = 28	80 × 1 = 80	248 × 1 = 248	776 × 1 = 776	2452 × 1 = 2452
	E1 (6.144)	unlimited	28 × 1 = 28	80 × 1 = 80	252 × 1 = 252	788 × 1 = 788	2488 × 1 = 2488
	E5	unlimited	16 × 1 = 16	49 × 1 = 49	154 × 1 = 154	487 × 1 = 487	1540 × 1 = 1540

**Table 17:** Total integration time $T_{\mathrm{IT}}=T_{C}\times N_{\mathrm{NC}}$ as function of the sensitivity and context with Galileo signals, for worst losses. Note that the sensitivities given are for the L1 C/A signal; those of E1 are 1.5 dB lower and those of E5 are 0.5 dB higher.

Context	Signal	$T_{C}$	−140 dBm	−145 dBm	−150 dBm	−155 dBm	−160 dBm
no assistance	E1 (4.096)	100 ms	100 × 1 = 100	100 × 2 = 200	100 × 10 = 1000	100 × 63 = 6300	100 × 520 = 52,000
	E1 (6.144)	100 ms	100 × 1 = 100	100 × 2 = 200	100 × 10 = 1000	100 × 64 = 6400	100 × 526 = 52,600
	E5	100 ms	100 × 1 = 100	100 × 2 = 200	100 × 6 = 600	100 × 30 = 3000	100 × 228 = 22,800
	E1 (4.096)	unlimited	100 × 1 = 100	200 × 1 = 200	600 × 1 = 600	1800 × 1 = 1800	5400 × 1 = 5400
	E1 (6.144)	unlimited	100 × 1 = 100	200 × 1 = 200	600 × 1 = 600	1800 × 1 = 1800	5500 × 1 = 5500
	E5	unlimited	100 × 1 = 100	200 × 1 = 200	400 × 1 = 400	1200 × 1 = 1200	3600 × 1 = 3600
assistance	E1 (4.096)	100 ms	100 × 1 = 100	100 × 2 = 200	100 × 8 = 800	100 × 48 = 4800	100 × 396 = 39,600
	E1 (6.144)	100 ms	100 × 1 = 100	100 × 2 = 200	100 × 8 = 800	100 × 49 = 4900	100 × 404 = 40,400
	E5	100 ms	100 × 1 = 100	100 × 1 = 100	100 × 4 = 400	100 × 22 = 2200	100 × 166 = 16,600
	E1 (4.096)	unlimited	44 × 1 = 44	140 × 1 = 140	432 × 1 = 432	1368 × 1 = 1368	4316 × 1 = 4316
	E1 (6.144)	unlimited	44 × 1 = 44	140 × 1 = 140	440 × 1 = 440	1388 × 1 = 1388	4384 × 1 = 4384
	E5	unlimited	28 × 1 = 28	86 × 1 = 86	272 × 1 = 272	858 × 1 = 858	2713 × 1 = 2713

**Table 18:** Ratio of total integration time (%) for different comparisons with Galileo signals. Top value is for average losses, and bottom value is for worst losses. Note that the sensitivities given are for the L1 C/A signal; those of E1 are 1.5 dB lower and those of E5 are 0.5 dB higher.

Comparison	Fixed Element	−140 dBm	−145 dBm	−150 dBm	−155 dBm	−160 dBm
Impact of using E5: E5 verses E1 4.096	$T_{C}=100$ ms (no assistance)	100	100	60	54	46
		100	100	60	48	44
	$T_{C}$ unlimited (no assistance)	100	100	75	70	68
		100	100	67	67	67
	$T_{C}=100$ ms (assistance)	100	100	50	47	43
		100	50	50	46	42
	$T_{C}$ unlimited(assistance)	57	61	62	63	63
		64	61	63	63	63
Impact of $T_{C}$: Unlimited verses 100 ms	E1 signal 4.096 (no assistance)	100	100	80	42	17
		100	100	60	29	10
	E1 signal 6.144 (no assistance)	100	100	80	40	17
		100	100	60	28	10
	E5 signal (no assistance)	100	100	100	54	26
		100	100	67	40	16
	E1 signal 4.096 (assistance)	28	80	62	41	18
		44	70	54	29	11
	E1 signal 6.144 (assistance)	28	80	63	41	18
		44	70	55	28	11
	E5 signal (assistance)	16	49	77	54	26
		28	86	68	39	16
Impact of assistance: Assistance verses no assistance	E1 signal 4.096 ($T_{C}=100$ ms)	100	100	80	79	77
		100	100	80	76	76
	E1 signal 6.144 ($T_{C}=100$ ms)	100	100	80	76	76
		100	100	80	77	77
	E5 signal ($T_{C}=100$ ms)	100	100	67	69	72
		100	50	67	73	73
	E1 signal 4.096 ($T_{C}$ unlimited)	28	80	62	78	79
		44	70	72	76	80
	E1 signal 6.144 ($T_{C}$ unlimited)	28	80	63	79	80
		44	70	73	77	80
	E5 signal ($T_{C}$ unlimited)	16	49	51	70	73
		28	43	68	72	75

**Table 19:** Ratio of complexity (product of processing time ratio and memory ratio) between the E1 signal with a sampling frequency of 6.144 MHz and the E1 signal with a sampling frequency of 4.096 MHz. Top rows do not consider the input memory, while bottom rows consider the input memory.

Sensitivity	Parallel without Assistance				Serial with Assistance			
	Non-Coherent		Coherent Only		Non-Coherent		Coherent Only	
	Average	Worst	Average	Worst	Average	Worst	Average	Worst
−140 dBm	3.0	3.0	3.0	3.0	3.0	3.0	3.0	3.0
−145 dBm	3.0	3.0	3.0	3.0	3.0	3.0	3.0	3.0
−150 dBm	3.0	3.0	3.0	3.0	3.0	3.0	3.0	3.1
−155 dBm	2.8	3.0	3.0	3.0	3.0	3.0	3.0	3.0
−160 dBm	3.0	3.0	3.0	3.1	3.0	2.8	3.0	3.0
−140 dBm	3.0	3.0	3.0	3.0	3.0	3.0	3.0	3.4
−145 dBm	3.0	2.9	3.0	3.2	3.0	3.8	3.0	3.8
−150 dBm	3.0	3.1	3.0	3.2	3.0	4.8	3.1	4.5
−155 dBm	2.9	4.3	3.0	3.5	3.0	7.2	3.1	5.0
−160 dBm	3.0	7.5	3.0	4.0	3.0	8.7	3.1	5.3

**Table 20:** Ratio of complexity (product of processing time ratio and memory ratio) between the E5 signal and the E1 signal with a sampling frequency of 4.096 MHz. Top rows do not consider the input memory, while bottom rows consider the input memory.

Sensitivity	Parallel without Assistance				Serial with Assistance			
	Non-Coherent		Coherent Only		Non-Coherent		Coherent Only	
	Average	Worst	Average	Worst	Average	Worst	Average	Worst
−140 dBm	41.9	41.9	41.9	41.9	10.5	10.5	5.4	6.2
−145 dBm	41.9	42.0	41.9	42.6	10.5	10.8	6.1	6.3
−150 dBm	43.3	43.5	32.3	27.6	10.9	10.9	6.7	6.8
−155 dBm	41.6	44.2	30.2	28.7	10.9	11.0	6.5	6.5
−160 dBm	44.3	44.0	29.1	27.6	10.9	11.2	6.5	6.5
−140 dBm	41.8	41.8	41.8	41.8	18.4	18.4	6.3	9.1
−145 dBm	41.8	41.2	41.2	45.5	18.4	18.4	8.8	12.7
−150 dBm	41.8	42.3	31.5	28.6	16.0	28.0	11.7	19.0
−155 dBm	35.4	42.5	28.4	30.3	17.4	40.7	13.9	23.5
−160 dBm	23.4	46.9	25.1	29.5	16.4	45.8	15.0	26.2

**Table 21:** Ratio of number of frequency bins between the E5 and E1 signals.

Sensitivity	Parallel without Assistance				Serial with Assistance			
	Non-Coherent		Coherent Only		Non-Coherent		Coherent Only	
	Average	Worst	Average	Worst	Average	Worst	Average	Worst
−140 dBm	0.8	0.8	0.8	0.8	0.8	0.8	0.4	0.5
−145 dBm	0.8	0.8	0.8	0.8	0.8	0.8	0.5	0.5
−150 dBm	0.8	0.8	0.6	0.5	0.8	0.8	0.5	0.5
−155 dBm	0.8	0.8	0.5	0.5	0.8	0.8	0.5	0.5
−160 dBm	0.8	0.8	0.5	0.5	0.8	0.8	0.5	0.5

**Table 22:** Final ratio of complexity (product of processing time ratio, memory ratio and frequency bins ratio) between the E5 and E1 signals obtained as the product of Table 10 and Table 11. Top rows do not consider the input memory, while bottom rows consider the input memory.

Sensitivity	Parallel without Assistance				Serial with Assistance			
	Non-Coherent		Coherent Only		Non-Coherent		Coherent Only	
	Average	Worst	Average	Worst	Average	Worst	Average	Worst
−140 dBm	31.4	31.4	31.4	31.4	7.9	7.9	2.3	2.9
−145 dBm	31.4	31.5	31.4	32.0	7.9	8.1	2.8	2.9
−150 dBm	32.5	32.6	18.2	13.8	8.1	8.2	3.1	3.2
−155 dBm	31.2	33.2	15.9	14.4	8.2	8.2	3.1	3.1
−160 dBm	33.3	33.0	14.8	13.8	8.2	8.4	3.1	3.1
−140 dBm	31.4	31.4	31.3	31.3	13.8	13.8	2.7	4.4
−145 dBm	31.4	30.9	31.3	34.1	13.8	13.8	4.0	5.9
−150 dBm	31.3	31.8	17.7	14.3	12.0	21.0	5.4	9.0
−155 dBm	26.5	31.9	14.9	15.1	13.0	30.5	6.5	11.1
−160 dBm	17.5	35.2	12.8	14.8	12.3	34.4	7.1	12.3

## Supplementary Materials

The spreadsheets used to obtain Table 3, Table 4, Table 5, Table 6, Table 7, Table 8, Table 9, Table 10, Table 11, Table 12, Table 13, Table 14, Table 15, Table 16, Table 17, Table 18, Table 19, Table 20, Table 21 and Table 22 excepted Table 13 are available online at http://www.mdpi.com/1424-8220/18/9/2779/s1.
